# Alcoholism and Psychiatric Disorders: Diagnostic Challenges

**Published:** 2002

**Authors:** Ramesh Shivani, R. Jeffrey Goldsmith, Robert M. Anthenelli

**Affiliations:** Ramesh Shivani, M.D., is an addiction psychiatry fellow; R. Jeffrey Goldsmith, M.D., is a clinical professor of psychiatry at and director of the Addiction Fellowships Program; and Robert M. Anthenelli, M.D., is an associate professor of psychiatry and director of the Addiction Psychiatry Division and of the Substance Dependence Program; all three at the University of Cincinnati College of Medicine, Cincinnati Veterans’ Affairs Medical Center, Cincinnati, Ohio

**Keywords:** AODD (alcohol and other drug dependence), diagnostic algorithm, diagnostic criteria, screening and diagnostic method for potential AODD, patient assessment, AODR (AOD related) mental disorder, behavioral and mental disorder, symptom, comorbidity, major depression, manic-depressive psychosis, personality disorder, anxiety, patient family history, medical history

## Abstract

Clinicians working with alcohol-abusing or alcohol-dependent patients sometimes face a difficult task assessing their patient’s psychiatric complaints because heavy drinking associated with alcoholism can coexist with, contribute to, or result from several different psychiatric syndromes. In order to improve diagnostic accuracy, clinicians can follow an algorithm that distinguishes among alcohol-related psychiatric symptoms and signs, alcohol-induced psychiatric syndromes, and independent psychiatric disorders that are commonly associated with alcoholism. The patient’s gender, family history, and course of illness over time also should be considered to attain an accurate diagnosis. Moreover, clinicians need to remain flexible with their working diagnoses and revise them as needed while monitoring abstinence from alcohol.

The evaluation of psychiatric complaints in patients with alcohol use disorders (i.e., alcohol abuse or dependence, which hereafter are collectively called alcoholism) can sometimes be challenging. Heavy drinking associated with alcoholism can coexist with, contribute to, or result from several different psychiatric syndromes. As a result, alcoholism can complicate or mimic practically any psychiatric syndrome seen in the mental health setting, at times making it difficult to accurately diagnose the nature of the psychiatric complaints (Anthenelli 1997; Modesto-Lowe and Kranzler 1999). When alcoholism and psychiatric disorders co-occur, patients are more likely to have difficulty maintaining abstinence, to attempt or commit suicide, and to utilize mental health services (Helzer and Przybeck 1988; Kessler et al. 1997). Thus, a thorough evaluation of psychiatric complaints in alcoholic patients is important to reduce illness severity in these individuals.

This article presents an overview of the common diagnostic difficulties associated with the comorbidity of alcoholism and other psychiatric disorders. It then briefly reviews the relationship between alcoholism and several psychiatric disorders that commonly co-occur with alcoholism and which clinicians should consider in their differential diagnosis. The article also provides some general guidelines to help clinicians meet the challenges encountered in the psychiatric assessment of alcoholic clients.

## Diagnostic Difficulties in Assessing Psychiatric Complaints in Alcoholic Patients

### A Case Example

A 50-year-old man presents to the emergency room complaining: “I’m going to end it all . . . life’s just not worth living.” The clinician elicits an approximate 1-week history of depressed mood, feelings of guilt, and occasional suicidal ideas that have grown in intensity since the man’s wife left him the previous day. The client denies difficulty sleeping, poor concentration, or any changes in his appetite or weight prior to his wife’s departure. He appears unshaven and slightly unkempt, but states that he was able to go to work and function on the job until his wife left. The scent of alcohol is present on the man’s breath. When queried about this, he admits to having “a few drinks to ease the pain” earlier that morning, but does not expand on this theme. He seeks help for his low mood and demoralization, acknowledging later in the interview that “I really don’t want to kill myself; I just want my life back to the way it used to be.”

The above case is a composite of many clinical examples observed across mental health settings each day, illustrating the challenges clinicians face when evaluating psychiatric complaints in alcoholic patients. The questions facing the clinician in this example include:

- Is the patient clinically depressed in the sense that he has a major depressive episode requiring aggressive pharmacological and psychosocial treatment?
- What role, if any, is alcohol playing in the patient’s complaints?
- How does one tease out whether drinking is the cause of the man’s mood problems or the result of them?
- If the man’s condition is not a major depression, what is it, what is its likely course, and how can it be treated?

As is usually the case (Anthenelli 1997; Helzer and Przybeck 1988), the patient in this example does not volunteer his alcohol abuse history but comes to the hospital for help with his psychological distress. The acute stressor leading to the distress is his wife’s leaving him; only further probing during the interview uncovers that the reason for the wife’s action is the man’s excessive drinking and the effects it has had on their relationship and family. Thus, a clinician who lacks adequate training in this area or who carries too low a level of suspicion of alcohol’s influence on psychiatric complaints may not consider alcohol misuse as a contributing or causative factor for the patient’s psychological problems.

In general, it is helpful to consider psychiatric complaints observed in the context of heavy drinking as falling into one of three categories—alcohol-related symptoms and signs, alcohol-induced psychiatric syndromes, and independent psychiatric disorders that co-occur with alcoholism. These three categories are discussed in the following sections.

### Alcohol-Related Psychiatric Symptoms and Signs

Heavy alcohol use directly affects brain function and alters various brain chemical (i.e., neurotransmitter) and hormonal systems known to be involved in the development of many common mental disorders (e.g., mood and anxiety disorders) (Koob 2000). Thus, it is not surprising that alcoholism can manifest itself in a broad range of psychiatric symptoms and signs. (The term “symptoms” refers to the subjective complaints a patient describes, such as sadness or difficulty concentrating, whereas the term “signs” refers to objective phenomena the clinician directly observes, such as fidgeting or crying.) In fact, such psychiatric complaints often are the first problems for which an alcoholic patient seeks help (Anthenelli and Schuckit 1993; Helzer and Przybeck 1988). The patient’s symptoms and signs may vary in severity depending upon the amounts of alcohol used, how long it was used, and how recently it was used, as well as on the patient’s individual vulnerability to experiencing psychiatric symptoms in the setting of excessive alcohol consumption (Anthenelli and Schuckit 1993; Anthenelli 1997). For example, during acute intoxication, smaller amounts of alcohol may produce euphoria, whereas larger amounts may be associated with more dramatic changes in mood, such as sadness, irritability, and nervousness. Alcohol’s disinhibiting properties may also impair judgment and unleash aggressive, antisocial behaviors that may mimic certain externalizing disorders, such as antisocial personality disorder (ASPD) (Moeller et al. 1998). (Externalizing disorders are discussed in the section “ASPD and Other Externalizing Disorders.”) Psychiatric symptoms and signs also may vary depending on when the patient last used alcohol (i.e., whether he or she is experiencing acute intoxication, acute withdrawal, or protracted withdrawal) and when the assessment of the psychiatric complaints occurs. For instance, an alcohol-dependent patient who appears morbidly depressed when acutely intoxicated may appear anxious and panicky when acutely withdrawing from the drug (Anthenelli and Schuckit 1993; Anthenelli 1997).

In addition to the direct pharmacological effects of alcohol on brain function, psychosocial stressors that commonly occur in heavy-drinking alcoholic patients (e.g., legal, financial, or interpersonal problems) may indirectly contribute to ongoing alcohol-related symptoms, such as sadness, despair, and anxiety (Anthenelli 1997; Anthenelli and Schuckit 1993).

### Alcohol-Induced Psychiatric Syndromes

It is clinically useful to distinguish between assorted commonly occurring, alcohol-induced psychiatric symptoms and signs on the one hand and frank alcohol-induced psychiatric syndromes on the other hand. A syndrome generally is defined as a constellation of symptoms and signs that coalesce in a predictable pattern in an individual over a discrete period of time. Such syndromes largely correspond to the sets of diagnostic criteria used for classifying mental disorders throughout the *Diagnostic and Statistical Manual of Mental Disorders, Fourth Edition* (DSM–IV) (American Psychiatric Association [APA] 1994) and its successor, the DSM–IV Text Revision (DSM–IV–TR) (APA 2000).

Publication of the DSM–IV marked the first time that clinicians could specifically diagnose several “alcohol-induced disorders” rather than having to lump alcohol-related conditions under the more generic rubric of an “organic mental syndrome” (Anthenelli 1997). Given the broad range of effects heavy drinking may have on psychological function, these alcohol-induced disorders span several categories of mental disorders, including mood, anxiety, psychotic, sleep, sexual, delirious, amnestic, and dementia disorders. According to the DSM–IV, the essential feature of all these alcohol-induced disorders is the presence of prominent and persistent symptoms, which are judged—based on their onset and course as well as on the patient’s history, physical exam, and laboratory findings—to be the result of the direct physiological effects of alcohol. To be classified as alcohol-induced disorders, these conditions also must occur within 4 weeks of the last use of or withdrawal from alcohol and should be of clinical significance beyond what is expected from typical alcohol withdrawal or intoxication (APA 1994).

The diagnostic criteria of the DSM–IV and DSM–IV–TR do not clearly distinguish between alcohol-related psychiatric symptoms and signs and alcohol-induced psychiatric syndromes. Instead, these criteria sets state more broadly that any alcohol-related psychiatric complaint that fits the definition given in the paragraph above and which “warrants independent clinical attention” be labeled an alcohol-induced disorder (APA 1994, 2000). In other words, alcohol-related psychiatric symptoms and signs can be labeled an alcohol-induced psychiatric disorder in DSM–IV or DSM–IV–TR without qualifying as syndromes.

Alcohol-induced psychiatric disorders may initially be indistinguishable from the independent psychiatric disorders they mimic. However, what differentiates these two groups of disorders is that alcohol-induced disorders typically improve on their own within several weeks of abstinence without requiring therapies beyond supportive care (Anthenelli and Schuckit 1993; Anthenelli 1997; Brown et al. 1991, 1995). Thus, the course and prognosis of alcohol-induced psychiatric disorders are different from those of the independent major psychiatric disorders, which are discussed in the next section.

### Alcoholism with Comorbid, Independent Psychiatric Disorders

Alcoholism is also associated with several psychiatric disorders that develop independently of the alcoholism and may precede alcohol use and abuse. These independent disorders may make certain vulnerable patients more prone to developing alcohol-related problems (Helzer and Przybeck 1988; Kessler et al. 1997; Schuckit et al. 1997*b*). One of the most common of these comorbid conditions is ASPD, an axis II personality disorder^1^ marked by a longstanding pattern of irresponsibility and violating the rights of others that generally predates the problems with alcohol. Axis I disorders commonly associated with alcoholism include bipolar disorder, certain anxiety disorders (e.g., social phobia, panic disorder, and post-traumatic stress disorder [PTSD]), schizophrenia, and major depression (Helzer and Przybeck 1988; Kessler et al. 1997). (Several of these common comorbid disorders are reviewed in detail in other articles of this journal issue.) It is important for clinicians to know which disorders are most likely to coexist with alcoholism so that they may specifically probe for these conditions when evaluating the patient’s complaints.

## Psychiatric Disorders Commonly Associated with Alcoholism

### Independent Major Depression

Mood disturbances (which frequently are not severe enough to qualify as “disorders”) are arguably the most common psychiatric complaint among treatment-seeking alcoholic patients, affecting upwards of 80 percent of alcoholics at some point in their drinking careers (Brown and Schuckit 1988; Anthenelli and Schuckit 1993). In keeping with the three broad categories described above into which such complaints may fall, mood problems may be characterized as one of the following:

- An expected, time-limited consequence of alcohol’s depressant effects on the brain
- A more organized constellation of symptoms and signs (i.e., a syndrome) reflecting an alcohol-induced mood disorder with depressive features
- An independent major depressive disorder coexisting with or even predating alcoholism.

When one applies these more precise definitional criteria and classifies only those patients as depressive who meet the criteria for a syndrome of a major depressive episode, approximately 30 to 40 percent of alcoholics experience a comorbid depressive disorder (Anthenelli and Schuckit 1993; Schuckit et al. 1997*a*).

Some controversy exists as to the precise cause-and-effect relationship between depression and alcoholism, with some authors pointing out that depressive episodes frequently predate the onset of alcoholism, especially in women (Kessler et al. 1997; Helzer and Przybeck 1988; Hesselbrock et al. 1985). Several studies found that approximately 60 percent of alcoholics who experience a major depressive episode, especially men, meet the criteria for an alcohol-induced mood disorder with depressive features (Schuckit et al. 1997*a*; Davidson 1995). The remaining approximately 40 percent of alcoholic women and men who suffer a depressive episode likely have an independent major depressive disorder—that is, they experienced a major depressive episode before the onset of alcoholism or continue to exhibit depressive symptoms and signs even during lengthy periods of abstinence.

In a study of 2,954 alcoholics, Schuckit and colleagues (1997*a*) found that patients with alcohol-induced depression appear to have different characteristics from patients with independent depressive disorders. For example, compared with patients with alcohol-induced depression, patients with independent depression were more likely to be Caucasian, married, and female; less experienced with other illicit drugs; less often treated for alcoholism; more likely to have a history of a prior suicide attempt; and more likely to have a family history of a major mood disorder.

### Bipolar Disorder

According to two major epidemiological surveys conducted in the past 20 years (Helzer and Przybeck 1988; Kessler et al. 1997), bipolar disorder (i.e., mania or manic-depressive illness) is the second-most common axis I disorder associated with alcohol dependence.^2^ Among manic patients, 50–60 percent abuse or become dependent on alcohol or other drugs (AODs) at some point in their illness (Brady and Sonne 1995). Diagnosing bipolar disorder in alcoholic patients can be particularly challenging. Several factors, such as the underreporting of symptoms (particularly symptoms of mania), the complex effects of alcohol on mood states, and common features shared by both illnesses (e.g., excessive involvement in pleasurable activities with high potential for painful consequences) reduce diagnostic accuracy. Bipolar patients are also likely to abuse drugs other than alcohol (e.g., stimulant drugs such as cocaine or methamphetamine), further complicating the diagnosis. As will be described in greater detail later, it can be helpful for an accurate diagnosis to obtain a careful history of the chronological order of both illnesses because approximately 60 percent of patients with both alcoholism and bipolar disorder started using AODs before the onset of affective episodes (Strakowski et al. 2000).

### Anxiety Disorders

Overall, anxiety disorders do not seem to occur at much higher rates among alcoholics than among the general population (Schuckit and Hesselbrock 1994). For example, results from the Epidemiologic Catchment Area survey indicated that among patients who met the lifetime diagnosis of alcohol abuse or dependence, 19.4 percent also carried a lifetime diagnosis of any anxiety disorder. This corresponds to only about 1.5 times the rate for anxiety disorders in the general population (Regier et al. 1990; Kranzler 1996). Specific anxiety disorders, such as panic disorder, social phobia, and PTSD, however, appear to have an increased co-occurrence with alcoholism (Schuckit et al. 1997*b*; Kranzler 1996; Brady et al. 1995).

As with alcohol-induced depression, it is important to differentiate alcohol-induced anxiety from an independent anxiety disorder. This can be achieved by examining the onset and course of the anxiety disorder. Thus, symptoms and signs of alcohol-induced anxiety disorders typically last for days to several weeks, tend to occur secondary to alcohol withdrawal, and typically resolve relatively quickly with abstinence and supportive treatments (Kranzler 1996; Brown et al. 1991). In contrast, independent anxiety disorders are characterized by symptoms that predate the onset of heavy drinking and which persist during extended sobriety.

### ASPD and Other Externalizing Disorders

Among the axis II personality disorders, ASPD (and the related conduct disorder, which often occurs during childhood in people who subsequently will develop ASPD) has long been recognized to be closely associated with alcoholism (Lewis et al. 1983). Epidemiologic analyses found that compared with nonalcoholics, alcohol-dependent men are 4–8 times more likely, and alcoholic women are 12–17 times more likely, to have comorbid ASPD (Helzer and Przybeck 1988; Kessler et al. 1997). Thus, approximately 15 to 20 percent of alcoholic men and 10 percent of alcoholic women have comorbid ASPD, compared with 4 percent of men and approximately 0.8 percent of women in the general population. Patients with ASPD are likely to develop alcohol dependence at an earlier age than their nonantisocial counterparts and are also more prone to having other drug use disorders (Cadoret et al. 1984; Anthenelli et al. 1994).

In addition to ASPD, other conditions marked by an externalization of impulsive aggressive behaviors, such as attention deficit hyperactivity disorder (ADHD) (Sullivan and Rudnik-Levin 2001), are also associated with increased risk of alcohol-related problems. (For more information on the relationship between alcoholism and ADHD, see the article by Smith and colleagues, pp. 122–129.)

## A Basic Approach to Diagnosing Patients with Alcoholism and Coexisting Psychiatric Complaints

Clinicians working in acute mental health settings often encounter patients who present with psychiatric complaints and heavy alcohol use. The following sections discuss one approach to diagnosing these challenging patients (also see the figure).

### Inquiring About Alcohol Use When Evaluating Psychiatric Complaints

As illustrated by the case example described earlier, patients seldom volunteer information about their alcohol use patterns and problems when they present their psychiatric complaints (Helzer and Przybeck 1988; Anthenelli and Schuckit 1993; Anthenelli 1997). Unless they are asked directly about their alcohol use, the patients’ denial and minimization of their alcohol-related problems lead them to withhold this important information, which makes assessment and diagnosis difficult. In addition, heavy alcohol use can impair memory, which may make the patient’s information during history-taking less reliable. Therefore, clinicians should gather information from several resources when assessing patients with possible alcohol-related problems, including collateral informants, the patient’s medical history, laboratory tests, and a thorough physical examination.

After obtaining a patient’s permission, his or her history should be obtained from both the patient and a collateral informant (e.g., a spouse, relative, or close friend). The information these collateral informant interviews yield can serve several purposes. First, by establishing how patterns of alcohol use relate to psychiatric symptoms and their time course, a clinician obtains additional information that can be used in the longitudinal evaluation of the patient’s psychiatric and alcohol problems, as described later. Second, by defining the role alcohol use plays in a patient’s psychiatric complaints, the clinician is starting to confront the patient’s denial, which is the patient’s defense mechanism for avoiding conscious analysis of the association between drinking and other symptoms. Third, by knowing that the clinician will be talking to a family member, the patient may be more likely to offer more accurate information. Fourth, if the patient observes that the clinician is interested enough in the case to contact family members, this may help establish a more trustful therapeutic relationship. Fifth, by involving family members early in the course of treatment, the clinician begins to lay the groundwork toward establishing a supporting network that will become an important part of the patient’s recovery program. Finally, the collateral informant can provide supplemental information about the family history of alcoholism and other psychiatric disorders that can improve diagnostic accuracy (Anthenelli 1997; Anthenelli and Schuckit 1993).

A review of the patient’s medical records is another potentially rich source of information. This review should look for evidence of previous psychiatric complaints or of laboratory results that might further implicate alcohol in the patient’s psychiatric problems (Allen et al. 2000). Pertinent laboratory results could include positive breath or blood alcohol tests; an elevation in biochemical markers of heavy drinking, such as the liver enzyme gamma-glutamyltransferase (GGT); and changes in the mean volume of the red blood cells (i.e., mean corpuscular volume), which also is an indicator of heavy drinking.

**Figure:**
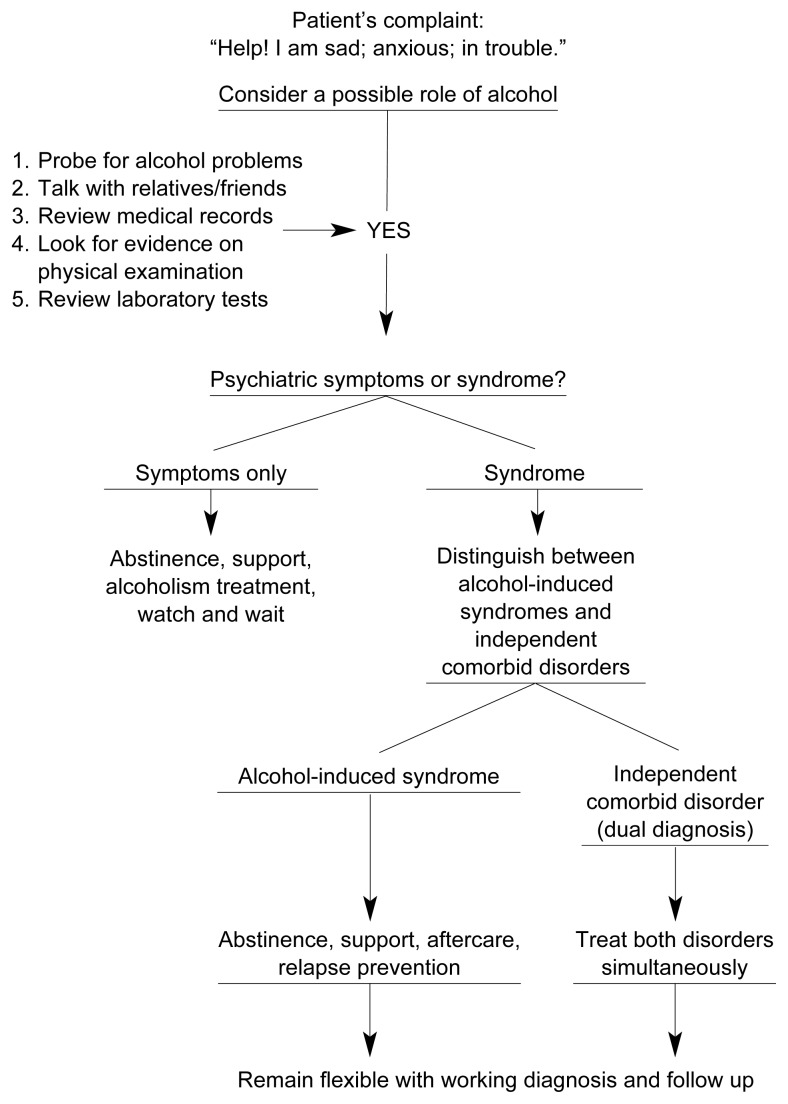
Schematic representation of a diagnostic algorithm for evaluating psychiatric complaints in patients for whom alcoholism may be a contributing factor. The algorithm helps the clinician decide if the compliants represent alcohol-induced symptoms, or an alcohol-induced syndrome that will resolve with abstinence, or an independent psychiatric disorder that requires treatment. SOURCE: Anthenelli 1997.

Laboratory tests, such as breathalyzer analyses or determination of blood alcohol concentrations, should also be performed to search for evidence of recent alcohol use that might aid in the assessment. These results also can provide indirect evidence of tolerance to alcohol (one of the diagnostic criteria of alcohol dependence) if the clinician documents relatively normal cognitive, behavioral, and psychomotor performance in the presence of blood alcohol concentrations that would render most people markedly impaired. Subsequent laboratory testing may also need to include other diagnostic procedures, such as brain imaging studies, to rule out indirect alcohol-related medical causes of the psychiatric complaints. For example, alcoholics suffering from head trauma might have hematomas (i.e., “blood blisters”) in the brain or other traumatic brain injuries that could cause psychiatric symptoms and signs (Anthenelli 1997).

Finally, all patients should undergo a complete physical examination. During this examination, the clinician should pay attention to physical manifestations of heavy alcohol use, such as an enlarged, tender liver. The combination of positive results on laboratory tests and physical examination points strongly to a diagnosis of alcohol abuse or dependence. This information can be used later on, when the physician presents his or her diagnosis to the patient and begins to confront the denial associated with the addiction (Anthenelli 1997).

### Differentiating Alcohol-Related Symptoms from Syndromic Mental Disorders

If the clinician suspects a diagnosis of alcoholism is appropriate, the next step is to evaluate the psychiatric complaints in this context. As mentioned earlier, alcohol produces its mind-altering and reinforcing effects by causing changes in the same neurotransmitter and receptor^3^ systems that are associated with most major psychiatric disease states. Partly as a result of these direct brain effects, heavy alcohol use causes psychiatric symptoms and signs that can mimic most major psychiatric disorders. These changes occur both in the absence and presence of alcohol, and during the initial assessment the clinician should determine when in the patient’s drinking cycle (i.e., during intoxication, acute withdrawal, protracted withdrawal, or stable abstinence for at least 3 months) these complaints are occurring.

Because heavy alcohol use can cause psychological disturbances, patients who present with co-occurring psychiatric and alcohol problems often do not suffer from two independent disorders (i.e., do not require two independent diagnoses). Therefore, the clinician’s job is to combine the data obtained from the multiple resources cited in the previous section and to establish a working diagnosis. It may be helpful to begin this process by differentiating between alcohol-related symptoms and signs and alcohol-induced syndromes. Thus, the preferred definition of the term “diagnosis” here refers to a constellation of symptoms and signs, or a syndrome, with a generally predictable course and duration of illness as outlined by DSM–IV.

Although heavy, prolonged alcohol use can produce psychiatric symptoms or, in some patients, more severe and protracted alcohol-induced psychiatric syndromes, these alcohol-related conditions are likely to improve markedly with abstinence. This characteristic distinguishes them from the major independent psychiatric disorders they mimic.

### Distinguishing Between Alcohol-Induced Syndromes and Independent Comorbid Disorders

Even after determining that a patient’s constellation of symptoms and signs has reached syndromic levels and warrants a diagnosis of a mood, anxiety, or psychotic disorder, the possibility remains that the patient has an independent comorbid disorder that may require treatment rather than an alcohol-induced syndrome that resolves with abstinence. Although some people experience more persistent alcohol-induced conditions (and some controversy remains over how to treat those patients), only clients with independent comorbid disorders should be labeled as having a dual diagnosis.

One approach to distinguishing independent versus alcohol-induced diagnoses is to start by analyzing the chronology of development of symptom clusters (Schuckit and Monteiro 1988). Using this technique as well as the DSM–IV guidelines, one can identify alcohol-induced disorders as those conditions in which several symptoms and signs occur simultaneously (i.e., cluster) and cause significant distress in the setting of heavy alcohol use or withdrawal (APA 1994). For example, a patient who exhibits psychiatric symptoms and signs only during recurrent alcohol use and after he or she has met the criteria for alcohol abuse or dependence is likely to have an alcohol-induced psychiatric condition. In contrast, a patient who exhibits symptoms and signs of a psychiatric condition (e.g., bipolar disorder) in the absence of problematic AOD use most likely has an independent disorder that requires appropriate treatment.

Establishing a timeline of the patient’s comorbid conditions (Anthenelli and Schuckit 1993; Anthenelli 1997), using collateral information from outside informants and the data obtained from the review of the medical records, may be helpful in determining the chronological course of the disorders. In this context the clinician should focus on the age at which the patient first met the criteria for alcohol abuse or dependence rather than on the age when the patient first imbibed or became intoxicated. This strategy provides more specific information about the onset of problematic drinking that typically presages the onset of alcoholism (Schuckit et al. 1995). If the clinician cannot determine exactly the time point when the patient met the criteria for abuse or dependence, this information can be approximated by determining when the patient developed alcohol-related problems that interfered with his or her life in a major way and affected the ability to function. Probing for such problems typically includes four areas—legal, occupational, and medical problems as well as social relationships. The age-at-onset of alcoholism then is estimated by establishing the first time that alcohol actually interfered in two or more of these major domains or the first time an individual received treatment for alcoholism. Further questioning should address whether the patient ever developed tolerance to the effects of alcohol or suffered from signs and symptoms of withdrawal when he or she stopped using the drug, both of which are diagnostic criteria for alcohol dependence.

After establishing the chronology of the alcohol problems, the patient’s psychiatric symptoms and signs are reviewed across the lifespan. The patient’s recollection of when these problems appeared can be improved by framing the interview around important landmarks in time (e.g., the year the patient graduated, her or his military discharge date, and so forth) and by the collateral information obtained. This method not only ensures the most accurate chronological reconstruction of a patient’s problems, but also, on a therapeutic basis, helps the patient recognize the relationship between his or her AOD abuse and psychological problems. Thus, this approach begins to confront some of the mechanisms that help the patient deny these associations (Anthenelli and Schuckit 1993; Anthenelli 1997).

While establishing this chronological history, it is important for the clinician to probe for any periods of stable abstinence that a patient may have had, noting how this period of sobriety affected the patient’s psychiatric problems. Using a somewhat conservative approach, such a probe should focus on periods of abstinence lasting at least 3 months because some mood, psychovegetative (e.g., altered energy levels and sleep disturbance), perceptual, and behavioral symptoms and signs related to AOD use can persist for some time. By using this timeline approach, the clinician generally can arrive at a working diagnosis that helps to predict the most likely course of the patient’s condition and can begin putting together a treatment plan.

### Considering Other Patient Characteristics

When evaluating the likelihood of a patient having an independent psychiatric disorder versus an alcohol-induced condition, it also may be helpful to consider other patient characteristics, such as gender or family history of psychiatric illnesses. For example, it is well established that women are more likely than men to suffer from independent depressive or anxiety disorders (Kessler et al. 1997).

Not surprisingly, alcoholic women are also more prone than alcoholic men to having independent mood or anxiety disorders (Kessler et al. 1997). Alcoholic women and men also seem to differ in the temporal order of the onset of these conditions, with most mood and anxiety disorders predating the onset of alcoholism in women (Kessler et al. 1997). Given these observations, it is especially important in female patients to perform a thorough psychiatric review that probes for major mood disorders (i.e., major depression and bipolar disorder) and anxiety disorders (e.g., social phobia).

Knowledge of the psychiatric illnesses that run in the patient’s family also may enhance diagnostic accuracy. For example, men and women with alcohol dependence and independent major depressive episodes have been found to have an increased likelihood of having a family history of major mood disorders (Schuckit et al. 1997*a*). Similar findings have been obtained for alcohol-dependent bipolar patients (Preisig et al. 2001). Thus, a family history of a major psychiatric disorder other than alcoholism in an individual may increase the likelihood of that patient having a dual diagnosis.

### Remaining Flexible with Diagnosis and Follow Up

Once a working diagnosis has been established, it is important for the clinician to remain flexible with his or her assessment and to continue to monitor the patient over time. Like most initial psychiatric assessments, the basic approach described here is hardly foolproof. Therefore, it is important to monitor a patient’s course and, if necessary, revise the diagnosis, even if improvement occurs with abstinence and supportive treatment alone during the first weeks of sobriety. The importance of continued followup for several weeks also is supported by empirical data showing that most major symptoms and signs are resolved within the first 4 weeks of abstinence. Therefore, unless there is ample evidence to suspect the patient has an independent psychiatric disorder, a 2- to 4-week observation period is usually advised before considering the use of most psychotropic medications.

### The Case Example Revisited

Recognizing that this was an emergency situation and that alcoholics have an increased rate of suicide (Hirschfeld and Russell 1997), the emergency room clinician admitted the patient to the acute psychiatric ward for an evaluation. The clinician also obtained the patient’s permission to speak with his wife. Despite the patient’s denial of alcoholism, this interview with a collateral informant corroborated the clinician’s suspicion that the man had long-standing problems with alcohol that dated back to his mid-20s. Laboratory tests showing an elevated GGT level supported the diagnosis. Moreover, a review of the patient’s medical records showed a previous hospitalization for suicidal ideation and depression 2 years earlier, after the patient’s mother had died.

The clinician then formulated a working diagnosis of probable alcohol-induced mood disorder with depressive features, based on three pieces of information. First, the patient had stated that his depression started about 1 week before admission, after his wife and family members confronted him about his drinking. This confrontation triggered a more intense drinking binge that ended only hours before his arrival in the emergency room. The patient complained of irritable mood and increased feelings of guilt during the past week, and he admitted he had been drinking heavily during that period. However, he denied other symptoms and signs of a major depressive episode during that period.

Second, the medical records indicated that the patient’s previous bout of depression and suicidal ideation had improved with abstinence and supportive and group psychotherapy during his prior hospitalization. At that time, the patient had been transferred to the hospital’s alcoholism treatment unit after 2 weeks, where he had learned some of the principles that had led to his longest abstinence of 18 months.

Third, both the patient and his wife said that during this period of prolonged abstinence the patient showed gradual continued improvement in his mood. He had worked an active 12-step program of sobriety and had returned to his job as an office manager.

During the first week of the current hospitalization, the patient’s suicidal ideation disappeared entirely and his mood gradually improved. He was transferred to the open unit and participated more actively in support groups. His denial of his alcoholism waned with persistent gentle confrontation by his counselors, and he began attending the hospital’s 12-step program. Three weeks after admission, he continued to exhibit improvement in his mood but still complained of some difficulty sleeping. However, he felt reassured by the clinician’s explanation that the sleep disturbance was likely a remnant of his heavy drinking that should continue to improve with prolonged abstinence. Nevertheless, the clinician scheduled followup appointments with the patient to continue monitoring his mood and sleep patterns.

## Summary

Alcohol abuse can cause signs and symptoms of depression, anxiety, psychosis, and antisocial behavior, both during intoxication and during withdrawal. At times, these symptoms and signs cluster, last for weeks, and mimic frank psychiatric disorders (i.e., are alcohol-induced syndromes). These alcohol-related conditions usually disappear after several days or weeks of abstinence. Prematurely labeling these conditions as major depression, panic disorder, schizophrenia, or ASPD can lead to misdiagnosis and inattention to a patient’s principal problem—the alcohol abuse or dependence. With knowledge of the different courses and prognoses of alcohol-induced psychiatric disorders, an understanding of the comorbid independent disorders one needs to rule out, an organized approach to diagnosis, ample collateral information, and practice, however, the clinician can improve diagnostic accuracy in this challenging patient population.

## Footnotes

1 The DSM–IV classifies mental disorders along several levels, or axes. In this classification, axis II disorders include personality disorders, such as ASPD or obsessive-compulsive disorder, as well as mental retardation; axis I disorders include all other mental disorders, such as anxiety, eating, mood, psychotic, sleep, and drug-related disorders.

2 The axis I disorders most commonly associated with alcoholism are other drug use disorders.

3 Receptors are protein molecules located on the surface of a cell that interact with extracellular signaling molecules, such as neurotransmitters and hormones, and convey that signal to the cell’s interior to induce the appropriate response.
